# A hierarchical approach to removal of unwanted variation for large-scale metabolomics data

**DOI:** 10.1038/s41467-021-25210-5

**Published:** 2021-08-17

**Authors:** Taiyun Kim, Owen Tang, Stephen T. Vernon, Katharine A. Kott, Yen Chin Koay, John Park, David E. James, Stuart M. Grieve, Terence P. Speed, Pengyi Yang, Gemma A. Figtree, John F. O’Sullivan, Jean Yee Hwa Yang

**Affiliations:** 1 Charles Perkins Centre, The University of Sydney, Sydney, NSW Australia; 2 School of Mathematics and Statistics, The University of Sydney, Sydney, NSW Australia; 3 Computational Systems Biology Group, Children’s Medical Research Institute, Westmead, NSW Australia; 4 Department of Cardiology, Royal North Shore Hospital, Sydney, NSW Australia; 5 Cardiovascular Discovery Group, Kolling Institute of Medical Research, The University of Sydney, Sydney, NSW Australia; 6 Faculty of Medicine and Health, The University of Sydney, Sydney, NSW Australia; 7 Heart Research Institute, Sydney, NSW Australia; 8 School of Life and Environmental Sciences, The University of Sydney, Sydney, NSW Australia; 9 School of Medical Sciences, University of Sydney, Sydney, Australia; 10 Imaging and Phenotyping Laboratory, Charles Perkins Centre, University of Sydney, Sydney, Australia; 11 Department of Radiology, Royal Prince Alfred Hospital, Camperdown, Australia; 12 Bioinformatics Division, Walter Eliza Hall Institute, Parkville, VIC Australia; 13 School of Mathematics and Statistics, University of Melbourne, Parkville, VIC Australia; 14 Department of Cardiology, Royal Prince Alfred Hospital, Sydney, NSW Australia; 15 Faculty of Medicine, TU Dresden, Germany

**Keywords:** Metabolomics, Mass spectrometry, Software, Statistical methods

## Abstract

Liquid chromatography-mass spectrometry-based metabolomics studies are increasingly applied to large population cohorts, which run for several weeks or even years in data acquisition. This inevitably introduces unwanted intra- and inter-batch variations over time that can overshadow true biological signals and thus hinder potential biological discoveries. To date, normalisation approaches have struggled to mitigate the variability introduced by technical factors whilst preserving biological variance, especially for protracted acquisitions. Here, we propose a study design framework with an arrangement for embedding biological sample replicates to quantify variance within and between batches and a workflow that uses these replicates to remove unwanted variation in a hierarchical manner (hRUV). We use this design to produce a dataset of more than 1000 human plasma samples run over an extended period of time. We demonstrate significant improvement of hRUV over existing methods in preserving biological signals whilst removing unwanted variation for large scale metabolomics studies. Our tools not only provide a strategy for large scale data normalisation, but also provides guidance on the design strategy for large omics studies.

## Abstract (web summary)

Mass spectrometry-based metabolomics is a powerful method for profiling large clinical cohorts but batch variations can obscure biologically meaningful differences. Here, the authors develop a computational workflow that removes unwanted data variation while preserving biologically relevant information.

## Introduction

Liquid chromatography–tandem mass spectrometry (LC–MS/MS) is a preferred method of metabolomic acquisition given its high sensitivity and dynamic range. Typically, a range of metabolites can be separated on a single high performance LC column and their relative abundance quantified in MS/MS. This enables capture of fingerprints of specific biological processes that are critical in precision medicine applications such as studying complex metabolic diseases, and discovering new therapeutic targets and biomarkers^1^. There are a number of large-scale cohort studies that have performed metabolomic analyses, such as the Consortium of Metabolomics Studies (COMETS)^2^, and the Framingham Heart Study (FHS)^3^.

Despite a rapid increase in the number of large-scale metabolomics studies, the normalisation of metabolomics data remains a key challenge^4^. Due to the data acquisition time of studies with a large sample size, prolonged study recruitment and potentially multiple samples at various time points for each participant, the data acquisition process may require the samples to be divided into multiple batches, and may span anywhere from months to years^4,5^. Signals often drift over extended periods due to multiple factors including buffer changes, pooled quality control (QC) sample solutions, instrument cleanliness, and machine scheduled maintenance^6^. Common intra-batch variations include changes in LC–MS/MS performance due to instrument-dependent factors such as component failure or inconsistency, and fouling of the column, LC or MS source. Common inter-batch variations include time-dependent instrument variations such as instrument cleaning, tuning, column change, or inconsistent sample preparation factors including change in equipment and operator. These technical factors have a substantial impact on downstream analytics and need to be appropriately accounted for to maximise the opportunity to identify true biological signals.

Several workflows have been designed for analysing metabolomics data (e.g. MetaboAnalyst^7^ and NormalyzerDE^8^). However, most of them adapt common normalisation methods developed for other omics platforms and do not account for signal drift across extended time or inter-batch variations which are distinct unwanted variations commonly observed in metabolomics studies. A number of metabolomics-specific normalisation methods have been developed in recent years. Many of these methods share conceptual similarities, such as regression-based methods^5,9–12^, machine learning approaches^13–15^ and other matrix factorisation approaches^16–18^. The common assumption behind the majority of these approaches is that the signal drift across extended time can be robustly estimated based on one specific sample alone, the pooled QC sample. This dependence on the pooled sample is suboptimal for several reasons: (1) even in the most careful hands, there will be batch variation in the thawing and extraction of the pooled sample; (2) long-term storage of plasma leads to variable stability of the metabolite pool, e.g. increases of amino acids, decreases in phosphatidylcholines^19^; (3) long-term storage leads to even greater changes in the protein pool^20^, which in turn variably influence metabolite levels; and (4) there are many logistical issues with long-term pool storage including refrigeration malfunction, sample loss, and sample exhaustion. Currently, there are no existing experimental designed strategies or methods to robustly account for normalisation over an extended period.

In this paper, we present an experimental sample arrangement strategy to embed biological sample replicates throughout large-scale experiments to facilitate the estimation of unwanted variation within and between batches with RUV-III^21^, which we will refer to as RUV in this paper. We propose a hierarchical approach to removing unwanted variation by harnessing information from sample replicates embedded in the sequence of experimental runs/batches and applying signal drift correction with robust linear or non-linear smoothers. An in-house targeted metabolomics study was performed on a hospital-based cohort of patients with atherosclerosis (BioHEART- CT) was conducted based on the proposed sample arrangement strategy, and we utilise this to assess the normalisation on a number of criteria including retention of biological signal, low variability among replication, and reproducibility of results in comparison to other existing methods. More specifically, we compare against the performance of a number of recently developed and commonly used methods in popular pipelines when applied to large cohort studies, such as Support Vector Regression (SVR)^5^, Systematic Error Removal using Random Forest (SERRF)^15^, and Removal of Unwanted Variation based approaches^22,23^ (Table 1). The hRUV method is accessible as an R package and also as a shiny application at https://shiny.maths.usyd.edu.au/hRUV/.

**Table 1:** List of existing normalisation methods.

Tags	Method	Resource/implementation
log2Raw_batch	RUV-III^21^	R package ruv version 0.9.7.1
MetNormalizer+SVR	Support vector regression	R package MetNormalizer^5^ version 1.3.02
NormalizeMets+RLSC	Robust locally estimated scatterplot smoothing^44^	R package NormalyzeMets^11^ version 0.24
SERRF	Systematic error removal using random forest^15^	Online: https://slfan.shinyapps.io/ShinySERRF/
NormalyzerDE_RLR	Global robust linear regression^12^	R package NormalyserDE^8^ version 1.7.0
NormalyzerDE_CycLoess	Cyclic loess^45^	
NormalyzerDE_VSN	Variance-stabilising normalisation^46^	
NormalyzerDE+GI	Global intensity	
NormalyzerDE+Quantile	Quantile normalisation^47^	
NormalyzerDE+mean	Mean^48^	
NormalyzerDE+median	Median^49^	
NormalyzerDE+log2	log_2_ transformation	
Ratio	Ratio^50^	

## Results

### Replicate arrangement strategy in large-scale metabolomics study

**Fig. 1:**
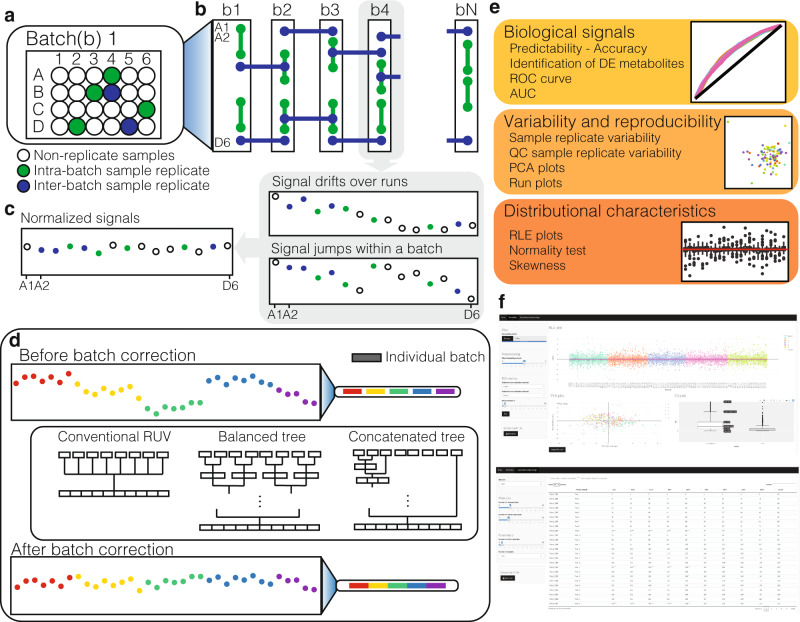
Schematic overview of the hRUV pipeline. **a** A schematic illustration of the plasma sample arrangement for the experimental batch in an array format where intra-batch sample replicates (green circles) and inter-batch sample replicates (blue circles) are embedded. **b** A schematic illustration of overall sample replicate design and arrangements. **c** Continuing the colour scheme from **b**, two illustrative run plots requiring intra-batch correction, with signal drift and other variations illustrated in the grey boxes. **d** A demonstration of signal variation before and after inter-batch correction in hRUV. A common approach to inter-batch correction is illustrated as conventional RUV and the proposed hierarchical approaches are illustrated. **e** A list of evaluation criteria to assess hRUV performances grouped into categories of biological signals, variability and reproducibility and distributional characteristics. **f** A screenshot of the user-friendly shiny application.

We developed a series of technical replications designed as a framework to enable effective data harmonisation in large cohorts studies over extended periods of time. Overall, 1000 samples were manually divided into 88 sample batches including 80 individual samples and 8 pooled samples per batch. Our design includes three types of replicates within each of 88 sample batches (in a 8 × 11 array format), these are the (i) classical pooled QC samples, (ii) single sample replicates in each row of a batch from a random selection of non-replicated samples in previous rows, which we call ‘short replicates’, and (iii) five randomly selected non-short replicated samples from each batch replicated to the next batch, which we call ‘batch replicates’. Figure 1a, b illustrates a schematic layout of the sample replicates design.

The classical pooled QC consists of a mixture of 10 μL of each of the 1002 samples, pooled together into a single tube. The pooled QC sample was aliquoted and frozen, and a fresh aliquot was thawed for each batch to minimise the impact of repeated freeze–thaw cycles. The spacing of the technical short replicates ~10 samples apart capture variation within a short time (~5 h, based on 30 min of run-time per sample). This is a good measure of the variation of the metabolomics experiment. In contrast to pooled QC samples, where one sample is repeated many times, short technical replicates are duplicates of different samples; this increase in heterogeneity of samples for the estimation of unwanted variation is more robust compared to estimation with pooled QC. Finally, the batch replicates measure the variation that occurs across different batches. These replicates are typically 60–70 samples apart, capturing variation over a longer time period of 48–72 h.

This design was used to generate data for a large metabolomics study consisting of 1002 individuals from the BioHEART-CT biobank and quantification of 100 metabolites per individual. After pre-processing, 53 metabolites were detected at adequate levels in plasma to be included in the analyses (Supplementary Data 1). The exact sample designs are given in Supplementary Data 2. In total, we had 164 replicates from one pooled QC sample, 230 duplicates from samples across 15 batches, and 140 batch duplicates from 70 samples. As expected, variation between replicates within a batch tends to be smaller compared to replicates between batches, as demonstrated in Supplementary Fig. 1. A shiny application was developed to enable easy generation of the replicate design upon input of the desired batch size and the desired number of inter-batch replicates. The experimental design with appropriate numbers and assignment of replicates inserted is then exported as a Comma Separated Values (CSV) file. The extra replicate tubes were prepared during the aliquoting and inserted into the appropriate positions during sample processing.

In the current study, we used 80 individual samples and 8 pooled samples per batch with consideration of blanks needed to be included on the autosampler sample tray (100 in capacity) as well as to minimise the overall number of pooled samples used. In practice, one could select any number of samples, subjects to the tray capacity of the autosampler as the unit for a batch. The notions of short and long (batch) replicates can be applied to any batch size to assess variation over a variety of distances.

### A hierarchical method to remove unwanted variation (hRUV) in large-scale omics experiments

To enable effective data harmonisation across large cohort studies or across an extended period of time, we propose a hierarchical approach to correct for the unwanted variation between smaller subsets of batches individually, and to sequentially expand to the next set of batches. The two key components of the hRUV can be summarised as follows: (i) signal drift correction within batches with a robust smoother that captures the irregular patterns affecting each metabolite as illustrated in Fig. 1c; and (ii) a scalable hierarchical approach to removal of unwanted variation between batches with the use of carefully assigned sample replicates.

The signal drift within each batch was corrected using a robust smoother that captures the trends visible by run order (Fig. 1c). We explored linear (robust linear model) and non-linear (local regression) model fitting smoothers to capture and remove the run order effects in the data. This is because, due to their chemical and physical properties (Supplementary Fig. 2a), each metabolite is affected differently across runs within each batch. These unique changes in signal for each metabolite need to be treated separately.

The concept of sequential batch correction is introduced here to enable scalability for large-scale cohort studies. This is a clear contrast to the conventional data integration for normalisation that involves estimating unwanted variation across all batches as a whole (Fig. 1d). Supplementary Fig. 2b shows the inter-batch variation and the differences in the corresponding adjustment factors over time, highlighting the need for dynamic normalisation. To this end, we propose two tree-structured approaches to estimating the different forms of unwanted variation across a large-scale cohort study, and to dynamically modify the batch effect removal across time. Figure 1d illustrates the two approaches: the balanced tree and the concatenating tree. The balanced tree approach requires log_2_(*n*) RUV adjustments to deal with *n* batches, while the concatenating tree approach requires *n* − 1 RUV adjustments. The concatenating approach requires more computation than the balanced tree approach but has an advantage when future integration with new batches is necessary. For once the initial batches are normalised, the additional RUV adjustments are needed are only as many as the number of new batches. While the balanced tree approach is quicker for large *n*, if *m* new batches are introduced in the future, the data will require additional log_2_(*n* + *m*) RUV adjustments from the individual batch level. Both approaches of hRUV normalisation method have been evaluated to assess retention of biological signal, low variability among replication, reproducibility and distributional characteristics (Fig. 1e).

The details of hRUV are included in the ‘Methods’ section. The final output of hRUV is a single normalised and batch-corrected matrix with all input matrices merged and ready for downstream analysis. The hRUV web application visualises diagnostic plots after normalisation and creates a downloadable file as a CSV matrix (Fig. 1f).

### Implementation of a smoother and RUV with sample replicates enables effective adjustment of within batch variation

We assess the performance of signal drift correction by comparing the results of smoothers against themselves and against the commonly used approaches (see ‘Methods’ section). Here, we applied both linear and non-linear smoothers to two sets of sample types; pooled QC samples, or all biological samples within a batch. In general, all four adjustment approaches (loess, rlm, loessSample, rlmSample) give adjusted values that have effectively removed any signal drift associated with experimental run order (Supplementary Fig. 3d–f). The intra-batch correction with all biological samples performs comparably to adjustments performed with pooled QC samples (Supplementary Fig. 3). This suggests a possible reduction in pooled QC samples during experimental design, and thus reducing the total run cost.

**Fig. 2:**
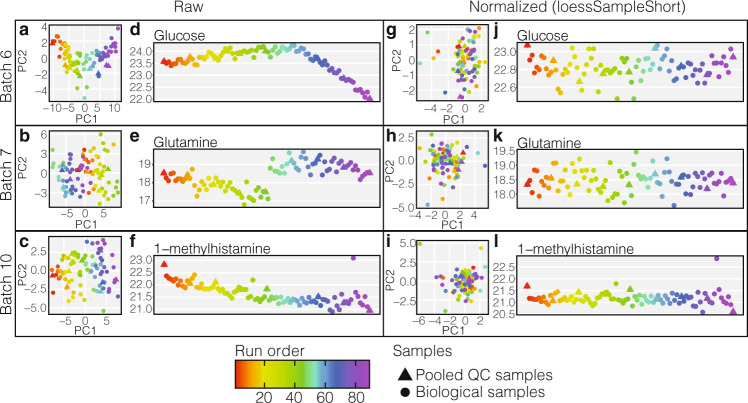
Examples of typical technical variations and signal drifts within each batch for different metabolites and comparison to normalised data. (Note vertical scale change.). **a**–**c** PCA plots of raw data in batches 6, 7 and 10 respectively, each marker is coloured by the sample run order. **d**–**f** Run plots exhibiting metabolite signal drift for glucose, glutamine, and 1-methylhistamine respectively, for their respective batches. **g**–**i** PCA plots from the same batches as **a**–**c** respectively with intra-batch normalised data using loessSampleShort method. **j**–**l** Run plot for the metabolites and batches illustrated in **d**–**f** but with intra-batch data normalised using loessSampleShort method. Source data are provided as a Source Data file.

In addition to using robust smoothers, the use of RUV with short replicates within each batch after a robust smoother further reduced the sample variations as demonstrated in Supplementary Figs. 2c and 4. Thus using a robust smoother and RUV with short replicates provides effective removal of various unwanted intra-batch variations (Fig. 2) and highlights the value of intra-batch sample replicates.

### hRUV is more effective in removing unwanted variation compared to other existing methods

**Fig. 3:**
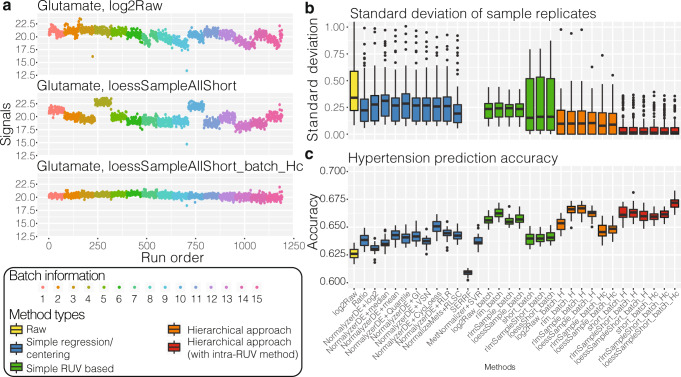
Key assessments of hRUV performances. **a** A run plot of raw, intra-batch-corrected and final hRUV normalised data in all 15 batches of the BioHEART-CT cohort. The *x* axis indicates the run order, the *y* axis indicates the signal of glutamate, and samples are coloured by the batch numbers. **b** Boxplots of all sample replicate (*n* = 175 sample replicates) standard deviations, where lower values indicate better performance. The boxes are coloured by the approach taken to normalise the data. The *y* axis of the plot is restricted to a range between 0 to 1 to highlight the differences between the majority of the methods. SERRF and MetNormalizer+SVR’s median sample replicate SD was >1 and thus is not shown. **c** Boxplots of hypertension prediction accuracies (*n* = 30 repeats) for all methods. Higher prediction accuracy indicates better performance. The colouring of the boxes are consistent with that in **b**. In **b**, **c**, the box indicates quartiles and the whiskers indicate the rest of the distribution, with outliers shown as dots. Source data are provided as a Source data file.

Across an extended period of time, there are many different types of unwanted variation. Figure 3a shows that across 1000 samples, we observe constant or irregular signal drift or abrupt jumps in signals. The run plots (Fig. 3a) illustrate the removal of technical variations introduced between batches and from run time effects for the metabolite glutamate.

Next, in comparison, we note that the hierarchical RUV normalisation was better at removing unwanted between-batch variation than the original single-step RUV. We compared the standard deviation (SD) between all sets of replicates, with lower values indicating better performance as the replicates should theoretically be identical. Figure 3b highlights lower SD between hierarchical normalisation methods (coloured in orange and red) and single-step ones (coloured in blue and green), suggesting that the hierarchical approach is more effective in batch correction across extended periods of time. Additionally, hierarchical approaches following intra-batch RUV (coloured red) showed even lower sample replicate variation.

### hRUV retains biological signals and outperforms existing normalisation methods

To examine the extent to which our method removes only unwanted variation and retains known biological signals, we performed supervised machine learning to illustrate our ability to identify known biological signals for disease prediction. Here we have chosen hypertension as a response variable and performed supervised machine learning classification to detect hypertension status from metabolomics abundance. We anticipate that a normalisation method that retains biological signals has a higher classification accuracy. The differential expression (DE) analysis to identify corresponding biomarkers (DE metabolites) measures the interpretability of the signal.

We observed that the average accuracy of hierarchical based normalisation methods was generally higher compared to one-step methods (Fig. 3c). The *loessAllShort_batch_Hc* method showed the best performance in prediction accuracy. This approach first adjusted for signal drift by fitting a loess line through all the samples and RUV corrected with short technical replicates within a batch, followed by applying RUV in a hierarchical fashion using batch technical replicates.

There are many metabolites reported to be associated with hypertension, including vasoactive metabolites (tryptophan and its derivatives), nitric oxide related metabolites (e.g. arginine, ADMA), microbiome-derived metabolites (short-chain fatty acids, trimethylamine-N-oxide), TCA cycle intermediates, ketone bodies, and bile acids^24^. While not all these metabolites were associated with hypertensive status in our cohort, we did see several biologically-plausible associations. The top-associated metabolite with hypertensive status in 1000 patients in hRUV normalised data was dimethylguanidino valeric acid (DMGV) ($\beta$ = 8.1, adjusted *P*-value = 2.3 × 10^−6^), whose role in cardiometabolic disease we previously discovered^25–27^. The second-most associated metabolite, cAMP ($\beta$= 4.4, adjusted *P*-value = 5.4 × 10^−5^), is a long-recognised second messenger in central, peripheral, and essential hypertension^28–30^. Although less well-established, proline is reported to elevate blood pressure: dietary studies showing association of intake of proline with blood pressure^31^, and preclinical studies demonstrating pressor actions of proline^32^. The hydroxylated form of proline, trans-4-hydroxyproline, was the third-most associated metabolite with hypertension in our cohort ($\beta$ = 2.4, adjusted *P*-value = 3.1 × 10^−^^4^). Together, these metabolites that are significantly associated with hypertension in our hRUV normalised data demonstrate the interpretability of the signal.

**Fig. 4:**
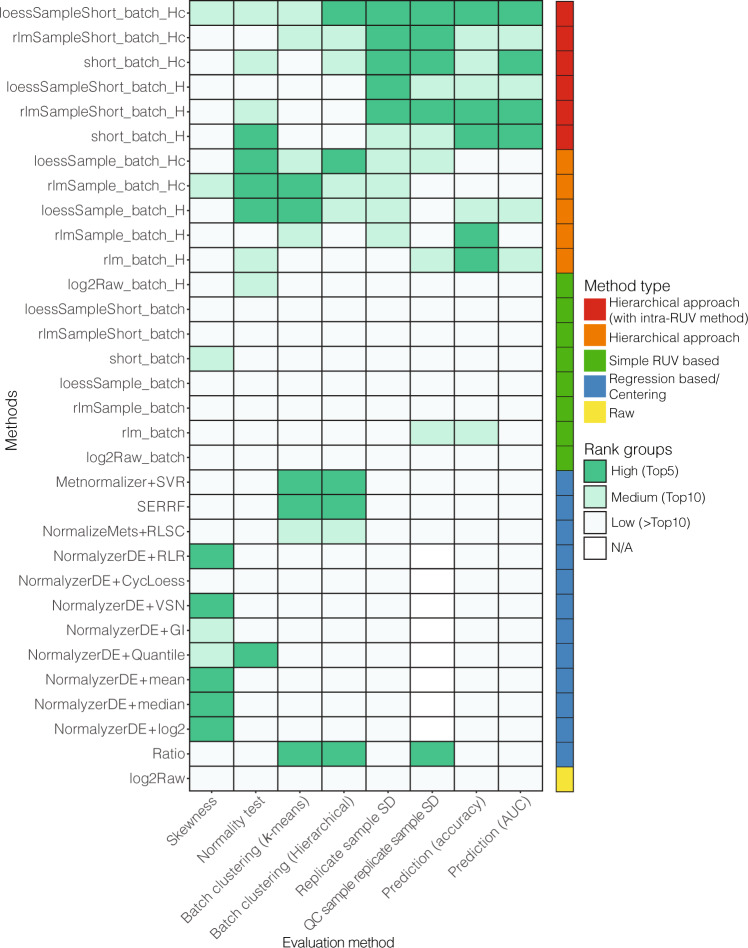
Heatmap of rankings in all evaluations criteria. The *y* axis represents all the methods explored in this study and the *x* axis represents all the qualitative evaluation metrics used for the evaluation of the integrated data. In each category, the evaluation scores are ranked and categorised into three groups, high, medium and low. The coloured bar on the right indicates the categorised method approaches consistent with Fig. 3b, c.

In general, we found that hRUV performed favourably in terms of maintaining strong biological signal and reducing unwanted variation such as signal drift and batch-specific noise in this large study (Fig. 4). Our evaluation metrics capture the trade-off between these two broad objectives. hRUV manages the trade-off between removing unwanted variation and retaining known clinical features of interest. We observed that the ratio, SVR, SERRF and RLSC methods have removed batch effects and reduced sample and pooled QC replicate variance, but as a trade-off, these methods result in a loss of biological signals, as evident by the low AUC and prediction accuracy values. Visualising all these quantities on a heatmap, we find that hRUV methods are ranked in the top 5–10 in most of the evaluation criteria (Fig. 4). The hRUV normalised data show the least variation across the different types of replicate samples and correctly removed batch driven technical noise, whilst maintaining a strong biological signal (Fig. 3b and Supplementary Figs. 5a, b, and 6).

### hRUV is robust to key decisions, types of hierarchical structure and choice of negative controls

We have investigated a number of parameters under the hRUV framework, including the various kinds of technical replication, types of negative control metabolite and types of hierarchical structure. We have explored different combinations of replication including pooled QC, inter-batch, and intra-batch replicates. We found that corrections with only pooled QC sample replicates over-estimates the unwanted variation and thus removes biological signals from the data (Supplementary Fig 7). This highlights the value of using sample replicates as opposed to the pooled QC samples in the estimation of intra- and inter-batch unwanted variation.

In contrast, the two hierarchical approaches in our hRUV show only a small differences in many of the evaluation measures. Both the balanced trees and the concatenating approaches performed adjustments between two sets of batches with 5 inter-batch replicates at each layer. As summarised in Fig. 4, the normalisation results are very similar between the two types of tree structure. We explored several approaches to obtaining negative controls for RUV, including a data-driven iterative procedure to select stable metabolites and the selection of all metabolites, and saw little difference between the normalised data outputs. We saw that the use of sample replicates demonstrated the greatest impact on the final normalised data (Supplementary Fig. 8). Finally, we have also demonstrated the scalability of hRUV using a large-scale public and simulated untargeted metabolomics data (Supplementary Figs. 9 and 10).

## Discussion

In this manuscript, we introduce a design strategy and present hRUV, a hierarchical approach to remove unwanted variation and batch correct large-scale metabolomics datasets, where there is a substantial unmet need. We have developed an R package and a shiny app for users to perform hRUV normalisation. We illustrated the performance of our method using metabolomics data derived from over 1000 patients in the BioHEART-CT biobank that was run over 15 batches across 44 days.

The careful arrangement of sample replicates on each batch is an important design consideration for large-scale mass-spectrometry studies. Here we believe that systematic arrangements perform better with hRUV normalisation than fully random ones. In our current design, the samples to be replicated were selected randomly from the previous batch and the corresponding repeats (batch replicates) were placed at the start of the current batch. Ideally, we would expect to select samples with this strategy whose positions were evenly distributed across the batch, but it is possible by chance to select samples whose positions are from only the first half or only the second half of the previous batch (Supplementary Data 2). This unintended clustering of selected samples was observed between batches 6 and 7, and also between batches 13 and 14, where replicates are selected only from the second half of the previous batch. This limits our ability to capture the unwanted variability across the whole batch, and as a result, we saw a slight shift in signal between these two batches for selected metabolites (Supplementary Fig. 5c).

While the proposed hRUV algorithm expects data without missing values, this is often not possible in large-scale metabolomics data due to the nature of the mass spectrometry technology and pragmatic issues related to real-world clinical studies. To this end, we include an option for users in which the missing values are first imputed prior to applying hRUV and the missing values can be replaced back after hRUV integration. This allows many more sparse metabolites to be incorporated for downstream analyses, which is an important aspect in large-scale metabolomics studies and may improve our chance of identifying novel metabolites from the data.

The negative controls are used in RUV to estimate the unwanted variation. The challenge with metabolomics is that the signals of the metabolites are dependent on their individual chemical properties^4,23^ and thus the selection of appropriate negative controls to correct for batch effect is a challenge. Whilst hRUV function accepts a user-defined set of negative controls, in our exploration of data-driven negative control metabolites compared to all metabolites as a negative control, we have found no significant differences between the two approaches in the removal of unwanted variation and utility of inter-batch sample replicates were more effective for batch correction (Supplementary Fig. 8).

In summary, hRUV uses sample replicates to integrate data from many batches in large-scale metabolomics studies. We show the value of suitably located sample replicates for estimating unwanted variation and guiding the design of future studies. While several other existing methods exist to correct large numbers of batches for intra-batch signal drift and inter-batch unwanted variation, hRUV performs consistently better than them in retaining biological variation whilst at the same time removing unwanted variation within and across batches.

## Methods

### Clinical samples

The samples used were from the BioHEART-CT discovery cohort, a study which has been described in detail previously^33^. The study was approved by the Northern Sydney Local Health District Human Research Ethics Committee (HREC/17/HAWKE/343) and all participants provided informed, written consent. Briefly, patients undergoing clinically indicated CT coronary angiogram for suspected coronary artery disease were recruited from multiple sites in Sydney, Australia. The inclusion criteria for BioHEART-CT study are patients aged 18 or older who had been referred for investigation of suspected CAD by CTCA, and who were willing and able to provide informed consent. Patients who were highly dependent on medical care who were unable to provide informed consent, as well as patients who were unwilling or unable to participate in ongoing follow-up were excluded. The BioHEART-CT discovery cohort utilised for this analysis included the first 1002 patients recruited to the BioHEART-CT study who had technically adequate CTCAs, sufficient stored blood samples for all planned biomarker discovery platforms, and who did not have a cardiac stent in situ or a prior history of coronary artery bypass surgery. Patients were advised to fast for 2 h prior to the procedure. 20–30 ml of venous blood was collected at the time of CTCA. After standard processing, plasma samples including replicates were aliquoted and stored at −80 °C until analysis.

Metabolites were extracted by mixing 10 μl plasma was mixed with 90 μl HILIC sample buffer, an acetonitrile: methanol: formic acid mix (75:25:0.2, v-v:v). The resulting solution was vortexed and spun at 14,000 rpm for 20 min to precipitate and remove plasma proteins. The metabolite containing supernatant (70 μl) was then transferred to a glass HPLC sample vial and resolved on an Agilent 1260 Infinity HPLC System with Atlantis Silica HILIC Column, (Waters, 100 Å, 3 µm, 2.1 mm × 150 mm) while *m*/*z* was determined by Qtrap5500 (Sciex)^25,34^ driven by Analyst software (Version 1.63, Build 8489, Sciex). Elution window and mass transitions used to identify eluted metabolites were pre-determined using pure compound (Supplementary Data 1). Each sample was eluted over a 25-min period using the gradient with mobile phase A (0.1% Formic acid (v/v%), 10 mM Ammonium Formate) and mobile phase B (0.1% Formic acid in Acetonitrile (v/v%) (Supplementary Table 1)). Each batch of samples took 40 h to complete. A total of 15 batches were completed over 44 days. Each metabolite was then manually integrated and determined using MultiQuant software (Version 3.03, Sciex) before the data were exported.

BioHEART-CT is an ongoing multi-platform imaging and biomarker discovery cohort. The results from initial analyses are currently under peer review with additional studies and analyses planned or underway. The data reported in this study is from the discovery cohort and has been approved by the study investigators. The current analysis is in line with this original approval and consent. BioHEART is a registered Australian and New Zealand Clinical Trial (ACTRN12618001322224).

### Technical replicate design

For each batch, three pooled QC samples were run to fine adjust the acquisition setting of the HPLC/MASS-Spec system, then a single pooled QC sample was repeated after every 10 runs. For each row of the 11 samples (10 individual samples + 1 pooled QC sample) from the 12th sample onwards (second row), we randomly selected a single biological sample from the previous row to be replicated at a random position in a current row (short replicate). For each batch, after the first three pooled QC samples, a random selection of 5 biological samples from the previous batch was repeated (batch replicate) and short replicates are embedded at each row. All randomisation was performed using the sample function in R^35^ [version 4.0.3]. The replicate design is available as a function ‘expDesign’ in the hRUV package, and also in our shiny application http://shiny.maths.usyd.edu.au/hRUV.

### Pre-processing of metabolomics data

Targeted metabolomics based on scheduled multiple reaction monitoring optimised to the metabolite of interest using authentic standards was applied in this study. Metabolite abundance peaks were integrated using the area under the curve for calibrated peaks from MultiQuant^TM^ version 3.0.3 (SCIEX), with manual peak integration was performed when necessary. This ensures the consistency of all the peaks integrated. The signal intensity of the ions were log_2_ transformed and metabolites that were not present in at least 50% of the samples were filtered out. Missing values were imputed using k-nearest neighbour with default parameters implemented in *DMwR2*^36^ package [version 0.0.2] in R^35^ [version 4.0.3]. We examined the three consecutive QC samples embedded at the start of each batch and removed any outlying measurements.

### Hierarchical approach to removal of unwanted variation (hRUV)

The hRUV algorithm was designed for experiments with a large number of batches. The process of hRUV consists of two key steps, including (i) within batch signal drift adjustment with robust smoothers and/or RUV; and (ii) the adjustment of the datasets with an unwanted variation using an RUV in a hierarchical approach. The main inputs to hRUV are raw signal matrix, with rows corresponding to metabolites and columns to samples as SummarizedExperiment^37^ [version 1.20.0] objects in R^35^ [version 4.0.3], a specific intra- and inter-batch normalisation method, the structure of the tree and the parameters for RUV. hRUV performs repeated RUV procedures to sequentially adjust the data over a large collection of batches, with the number of unwanted variation factors (*k*) defaulting to 5. Further details of the workflow is described in Supplementary Note 1.

*Part I: Signal drift adjustment within a batch*

In the present setting, batch refers to one 88 sample run. However, this can be any pre-specified number of samples. After pre-processing of metabolomics data, hRUV begins intra-batch normalisation by fitting a linear (or alternatively a non-linear) smoothers. An optional RUV step is then applied using sample replicates when specified by the user.

(i) Standard adjustment (ratio): The signal ratio was calculated by dividing the sample signal by the signal of its nearest pooled QC sample run. Let us denote ${P}_{L}$ as the early run pooled QC sample at run *L* and ${P}_{L+M}$ by the next pooled QC sample in a batch at run *L* + *M*, where $M$ denotes the number of runs between ${P}_{L}$ and ${P}_{L+M}$. Then the signal ratio is defined as follows:

1 $${{{{{\rm{If}}}}}}\,L \, < \,l\,\le\, L+\frac{M}{2},\\ \hat{s}_{il}=\frac{{s}_{il}}{{P}_{L}}$$

2 $${{{{{\rm{else}}}}}}\,{{{{{\rm{if}}}}}}\,L+\frac{M}{2} \, < \, l\,\le\, L+M,\\ \hat{s}_{il}=\frac{{s}_{il}}{{P}_{L+M}}$$

where ${s}_{il}$ denotes a signal of a sample with metabolite *i* at run number *l*.

(ii) Loess line: A loess line was fitted to all the biological samples for each metabolite within a batch with the default span parameter of 0.75. The differences between the fitted line to the median of each metabolite across all samples per batch were calculated for adjustment of each samples as follows:

3 $${\hat{y}_{ij}}={y}_{ij}+({\tilde{y}}_{i}-\hat{y}_{ij}^{\ast })$$

where $\,{y}_{ij}$ represents a log_2_ transformed signal for sample *j* in metabolite $i$ in a batch and $\hat{y}_{ij}^{\ast }$ denote a loess fitted value of ${y}_{ij}$ and $\tilde{{y}_{i}}$ denote the median of ${y}_{ij}$ for all *j*. Here, the loess line uses the loess function in the *stats*^35^ [version 4.0.3] package.

(iii) Linear line: A robust linear model (rlm) against the run index was fitted to the log_2_ transformed signal using the rlm function from the *MASS*^38^ [version 7.3-53.1] package for all the biological samples, with maximum number of iterations set to 100. The adjustments to each sample were calculated as with to the loess approach where $\hat{y}_{i}^{\ast }$, denotes the predicted value of ${y}_{i}$.

(iv) Loess line fitting with pooled QC samples: Similar to (ii), we fit the loess line to the pooled QC samples only. The adjusted value for each sample were calculated using the predicted values from the model ($\hat{y}_{pij}^{\ast }$).

4 $${\hat{y}_{ij}}={\hat{y}_{ij}}+({\tilde{y}}_{i}-\hat{y}_{pij}^{\ast })$$

(vii) Linear line fitting with pooled QC samples: Likewise, we fit the rlm to the pooled QC samples. The adjusted values for each sample were calculated using the predicted values from the model.

(viii) RUV based approaches: We incorporated sample replicates into the design matrix of RUV introduced by Molania et al.^21^. These sample replicates are utilised to estimate the unwanted variation as the signals of these replicate samples should theoretically be identical. All metabolites were used as the negative controls for RUV and the number of unwanted factors to use (*k*) was taken to be 5.

*Part II: Hierarchical batch integration design*

After intra-batch normalisation is complete, hRUV performs batch correction of multiple batches using either the balanced tree or the concatenating approach.

(i) Balanced tree. The balanced tree approach to normalisation removes unwanted variation between pairs of batches at different levels of the tree. In this approach, we begin by removing unwanted variation between pairs of neighbouring batches. In the next layer of adjustment, we pair the two neighbouring groups of integrated batches (sets of 2 batches) and repeated the process to expand the number of batches per set until the last layer, where we have a single group of all the normalised, as illustrated in schematics in Fig. 1d. For a study with *n* batches, this will requires log_2_(*n*) RUV adjustments.
(ii) Concatenation. As with the balanced tree approach, the concatenating approach removes unwanted variation between pairs of batches, but sequentially. We begin with the first two batches, and sequentially introduce subsequent batches, removing unwanted variation as illustrated in schematics in Fig. 1d. For a study with *n* batches, this will require *n−*1 number of RUV adjustments.

For both the balanced tree and the concatenation methods, we apply RUV at each layer as follows:

Let us denote by *B* the pair of batches of interest, *M* as the number of metabolites, and *S* as the number of samples in *B*. The mean adjusted sample ${Z}_{{{{{{\mathrm{mbc}}}}}}}$ can be calculated as:

5 $${Z}_{{{{{{\mathrm{mbs}}}}}}}={Y}_{{{{{{\mathrm{mbs}}}}}}}-{Y}_{m{{{{\mathrm{.}}}}}.},$$

where ${Y}_{m{{{{\mathrm{}}}}}..}$ is the average expression of metabolite *m* across samples *S* and batches *B* calculated by:

6 $${Y}_{m{{{{\mathrm{.}}}}}.}=\frac{1}{S}\mathop{\sum}\limits_{bs}{Y}_{{{{{{\mathrm{mbs}}}}}}};$$

The mean adjusted data ${Z}_{S\times M}$ can be fitted to the model underlying the RUV model, which is formulated as:

7 $${Z}_{S\times M}={X}_{S\times p}{\beta }_{p\times M}+{W}_{S\times k}{\alpha }_{k\times M}+{\in }_{S\times M}$$

where *X* is the matrix of factor of interest; *p* is the number of factors of interest; *W* is the unobserved design matrix corresponding to the unwanted factors; *k* is the linear dimension of the unwanted factors, which is unknown; and $\in$ denotes the random error. Thus, the RUV normalised data can be represented as:

8 $${\hat{Z}}_{S\times M}={Z}_{S\times M}-{\hat{W}}_{S\times k}{\hat{\alpha }}_{k\times M}$$

After the RUV, ${Y}_{m{{{{\mathrm{}}}}}..}$ is returned back to the mean adjusted RUV normalised data as follows:

9 $${\hat{Z}}_{mbs}^{\ast }={\hat{Z}}_{mbs}+{Y}_{m{{{{\mathrm{.}}}}}.}$$

### Data-driven negative metabolite selection

We explored an adaptive data-driven selection of negative control metabolites in comparison to the use of all metabolites in an RUV method. Adaptive selection is performed by ranking non-differentially expressed metabolites by *P*-values per batch for the hypertension response variable. We carried out this analysis with the *limma*^39^ [version 3.46] package in R^35^ [version 4.0.3].

### Performance evaluation/evaluation metric processing

We evaluate hRUV methods including 13 publicly available metabolomics data normalisation methods (Table 1). Details of the method abbreviations are explained in Table 2. These packages were installed either through the official CRAN or Bioconductor website where available, or from GitHub pages. For all 13 existing methods, we used the default settings and parameters as described in the package README or vignette for training each model.

**Table 2:** A normalisation method abbreviation dictionary.

Tags	Definition
X_Y	Methods separated by ‘_’ indicates 2 levels of adjustments applied. In this example, X is the intra-batch adjustment applied and Y indicates the inter-batch adjustment method applied
X+Y	Methods separated by ‘+’ denotes a method Y implemented in an R package X
loess	A loess line fitting method with pooled QC samples
rlm	A robust linear model fitted to pooled QC samples
loessSample	A loess line fitting method only on biological samples
rlmSample	A robust linear model fitted only on biological samples
short	RUV with short (intra-batch) sample replicates
batch	RUV with batch (inter-batch) sample replicates
_H	A hierarchical balanced tree approach
_Hc	A hierarchical concatenating tree approach

### Evaluation metrics and plots

(i) Skewness: The skewness of samples were calculated with skewness function from *e1071*^40^ [version 1.7-4] package in R^35^ [version 4.0.3].

Let us denote ${x}_{j}$ for the non-missing elements of **x**, *n* for the number of samples, $\mu$ for the sample mean, *s* for the sample standard deviation, and ${m}_{r}=\mathop{\sum}\limits_{j}{({x}_{j}-\mu )}^{r}/n$ for the sample moments of order *r*. The skewness then can be calculated as:

10 $${{{{{\rm{Skewness}}}}}}=\frac{{m}_{3}}{{s}^{3}}$$

(ii) Normality metric: The normality tests were performed with Shapiro–Wilk normality test implemented in shapiro.test function from the *stats*^35^ [version 4.0.3] package in R^35^ [version 4.0.3].

(iii) Predictability with accuracy: To assess the predictability of a normalised dataset, we utilised a binary diagnosis of hypertension as the response variable. This was chosen as it had a reasonably balanced class distribution, as 39% of the cohort had hypertension. We used a Support Vector Machine (SVM) implemented in the *e1071*^40^ [version 1.7-4] package to predict the hypertension status of participants of the study. We measured the average accuracy via a 30-repeated 10 folds cross-validation strategy.

(iv) Signal strength with AUC: We use the same prediction model from (iii) and calculate the area under the ROC curve (AUC) values.

(v) Standard deviation of replicates (SD replicates): To demonstrate the variation between the replicate samples after normalisation, for each set of replicated sample, we calculated the standard deviation for each metabolite and visualised the results as a boxplot. A low standard deviation indicates a small variability between the replicates and thus illustrates that the replicates are close to identical.

(vi) Clustering by batch (Reduction in batch effect): To assess the removal of batch effects, we performed unsupervised hierarchical and *k-*means clustering (hclust and kmeans in *stats*^35^ [version 4.0.3] package in R^35^ [version 4.0.3] respectively) where we set the number of clusters *k* to the number of batches. The cluster output is evaluated using adjusted rand index (ARI):

11 $${{{{{\rm{ARI}}}}}}=\frac{2(ad-bc)}{(a+b)(b+d)+(a+c)(c+d)},$$

where $a$ is the number of pairs of samples partitioned into the same batch group by the clustering method, $b$ is the number of pairs of samples partitioned into the same cluster but does not belong to the different batch group, $c$ is the number of pairs of samples partitioned into different clusters but belongs to the same batch group and $d$ is the number of pairs of samples correctly partitioned into different clusters. A low ARI value indicates lower concordance with the batch information and thus demonstrates the removal of a batch effect in the data.

(vii) Differential expression (DE) analysis of hypertension: To assess the biological signal in the normalised data, we performed DE analysis with the R package *limma*^39^ [version 3.46.0]. We identified a set of metabolites with a 5% level of significance and verified their association with hypertension from the literature.

### Diagnostic plots

To graphically assess whether the normalisation method or the choice of parameters of hRUV has effectively corrected the batch effect, we have provided three kinds of diagnostic plots: (1) PCA plots; (2) relative log expression (RLE) plots^41^; (3) metabolite run plots.

#### PCA plots

PCA plots were generated using all metabolites. We show the first and second principal components.

#### Relative log expression (RLE) plots

RLE plots are a useful tool to visualise unwanted variation. RLA plots are boxplots of RLA for each sample, calculated as ${Y}_{ij}-{\tilde{Y}}_{i}$, where ${\tilde{Y}}_{i}$ = median{${Y}_{ij}:j=1,2,\,\ldots$}, and ${Y}_{ij}\,$ is the log signal value of metabolite *i* in sample *j*. The samples from different batches should have a similar distribution, and the medians of the boxplots should be close to zero if the unwanted variations are removed.

#### Metabolite run plots

Metabolite plots are a useful diagnostic visualisation to visualise the signal drifts. The run plots are a scatter plot of signals for each metabolite against the run order of all the samples. The overall shape of the scatter plot should be a flat horizontal bar. All other shapes of trend in the scatter plot is an indication of signal drift.

### Reporting summary

Further information on research design is available in the Nature Research Reporting Summary linked to this article.

## Supplementary information

Supplementary Information

Description of Additional Supplementary Files

Supplementary Data 1

Supplementary Data 2

Reporting summary

## Data Availability

The raw metabolomics data generated in this study have been deposited in the MetaboLights under accession code MTBLS2483^42^. The processed metabolomics data are available at https://github.com/SydneyBioX/BioHEART_metabolomics. The sample order design data generated in this study are provided in Supplementary Data 2. Source data are provided with this paper.

## Source data

Source Data

## Code availability

The hRUV implementation is available as an R package stored at https://github.com/SydneyBioX/hRUV and Zenodo^43^, and as a web shiny application available via https://shiny.maths.usyd.edu.au/. A typical workflow of hRUV is demonstrated on the hRUV package web page (https://sydneybiox.github.io/hRUV/). We have set an upload limit for the current shiny application and clarified the capability and file size limitation on the website. We believe the current limit is sufficient for most datasets, including much larger untargeted metabolomics datasets. We recommend installing our hRUV R package locally for users with a much larger dataset to run on their computer or server.
