# Supplementary material for: A hierarchical approach to removal of unwanted variation for large-scale metabolomics data
Source: Nat Commun. 2021 Aug 17;12:4992. doi: 10.1038/s41467-021-25210-5 (PMC8371158; doi:10.1038/s41467-021-25210-5)
Supplement: Supplementary file 1 — Supplementary Information [file 41467_2021_25210_MOESM1_ESM.pdf]

Supplementary Information

A hierarchical approach to removal of unwanted variation for large-scale metabolomics data

Taiyun Kim[1,2,3], Owen Tang[1,4,5,6], Stephen T Vernon[1,4,5,6], Katharine A Kott[1,4,5,6], Yen Chin Koay[1,6,7], John Park[1,4,5,6], David James[1,8], Stuart Grieve[1,5,9,10], Terence P Speed[11,12], Pengyi Yang[1,2,3,6], Gemma A Figtree[1,4,5,6], John F. O'Sullivan[1,6,7,13,14], Jean Yee Hwa Yang[1,2,*]

Supplementary Figures

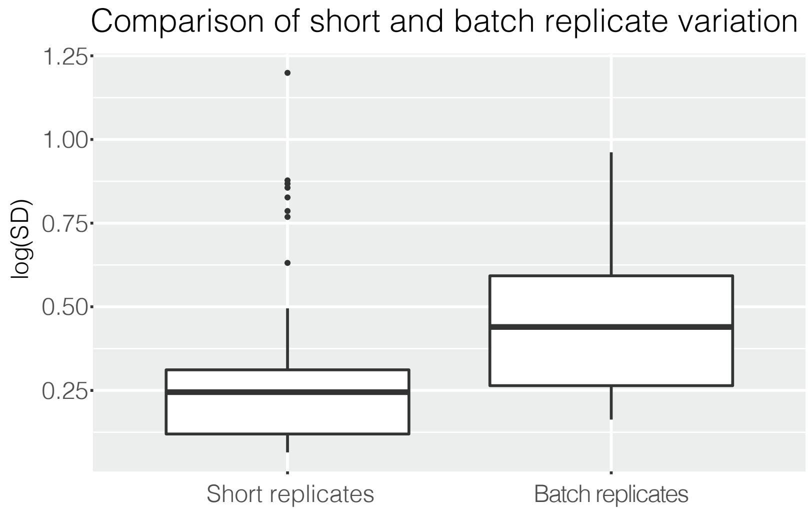

Supplementary Figure 1. **A comparison of short and batch replicate standard deviations.** Boxplots comparison of intra- (short, n_{short}=115 sample replicates) and inter- (batch, n_{batch}=68 sample replicates) replicates standard deviations (SD). The y-axis is a natural log transformed SD. The batch replicate samples exhibit higher standard deviations than the short sample replicates. The box indicates quartiles and the whiskers indicate the rest of the distribution, with outliers shown as dots. Source data are provided as a Source data file.

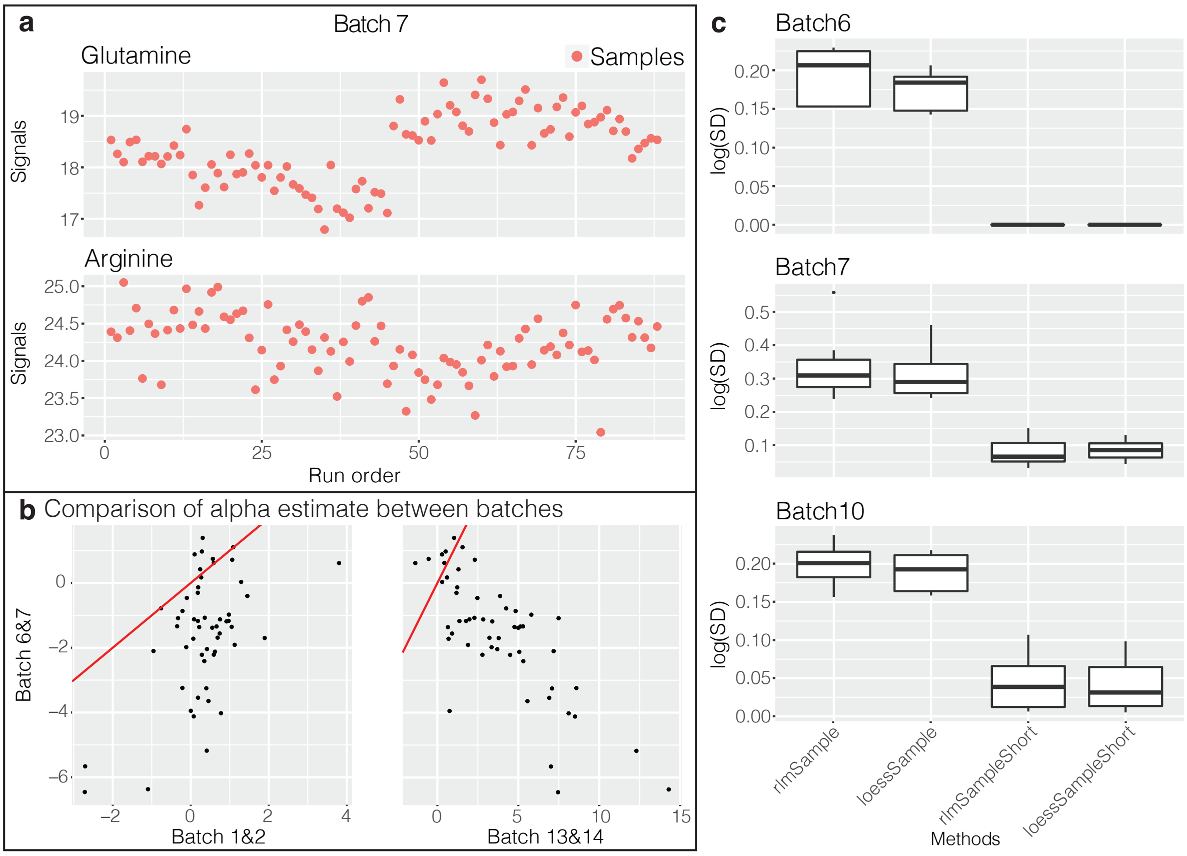

Supplementary Figure 2. An overview of different intra-batch and inter-batch noise. a A run plot of raw data values from batch 7 exhibiting signal drift in the glutamine and arginine measurements. **b** A comparison of the first component of the estimated alpha term in the intra-batch RUV correction across two adjacent pairs of batches. The y-axis shows the alpha values estimated from batches 6 and 7. The x-axis shows the alpha values estimated from batches 1 and 2 (left) and also from batches 13 and 14 (right). The red line is y=x. **c** Boxplots of sample replicate (*n*=5 sample replicates in all batches and for all methods) standard deviations for the robust smoother with and without RUV using short replicates. The y-axis is a natural log transformed SD. In **c**, the box indicates quartiles and the whiskers indicate the rest of the distribution, with outliers shown as dots. Source data are provided as a Source data file.

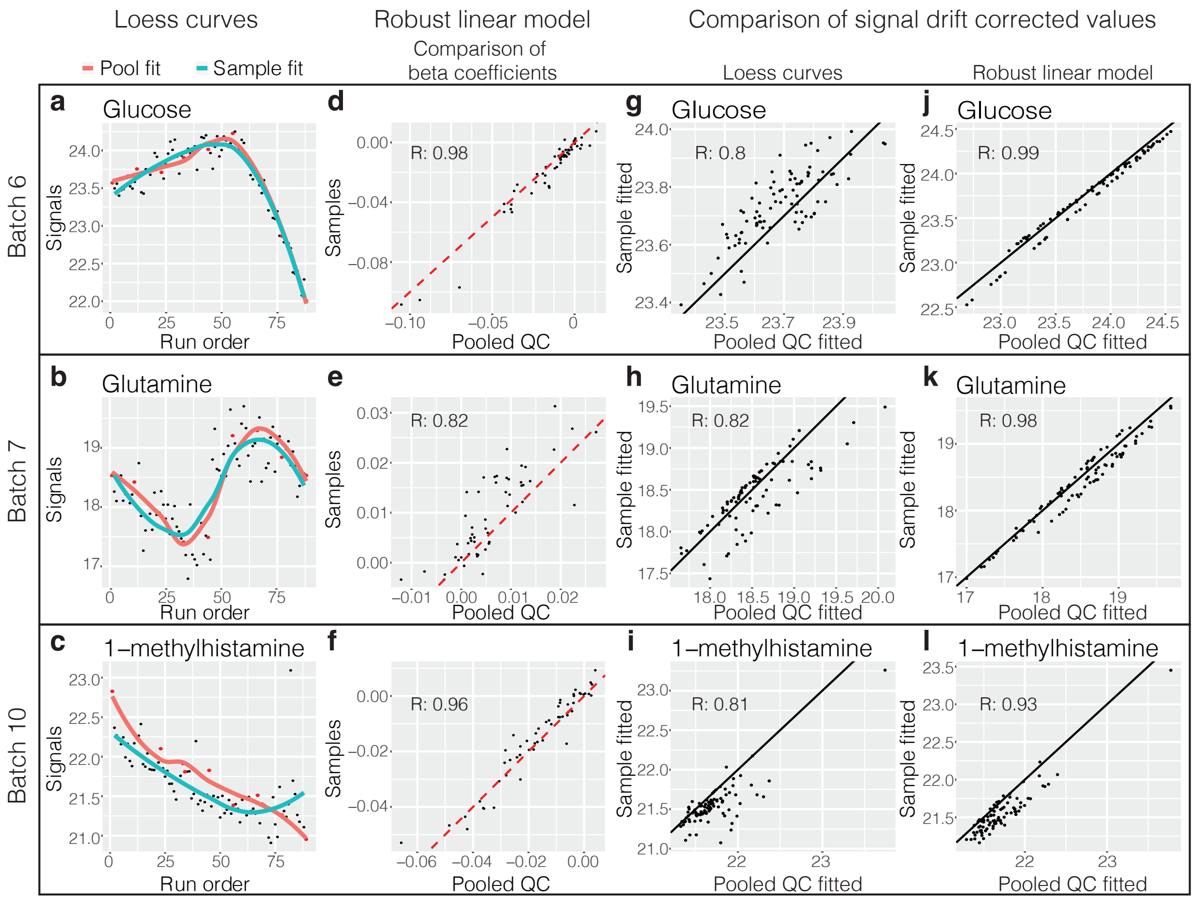

Supplementary Figure 3. A comparison of robust smoothers fitted to all samples versus to pooled QC samples. Comparisons of pooled QC fitted smoother and sample fitted smoothers. The rows of the panel exhibit data from Batch 6, 7 and 10 samples. The columns exhibit types of comparisons. **a-c** A pooled QC fitted loess curves (red) and sample fitted loess curves (blue) of glucosePos2 in Batch6, glutamine in Batch 7 and 1-methyl histamine in Batch 10 respectively, all against run order. **d-f** Beta coefficients of sample fitted robust linear model (y-axis) against pooled QC fitted robust linear model (x-axis). **g-i** Signal drift adjusted values from sample fitted loess smoother (y-axis) against pooled QC fitted values (x-axis). **j-l** Similarly to **g-i**, with signal drift adjusted values from pooled QC fitted (x-axis) and sample fitted (y-axis) robust linear models. Source data are provided as a Source data file.

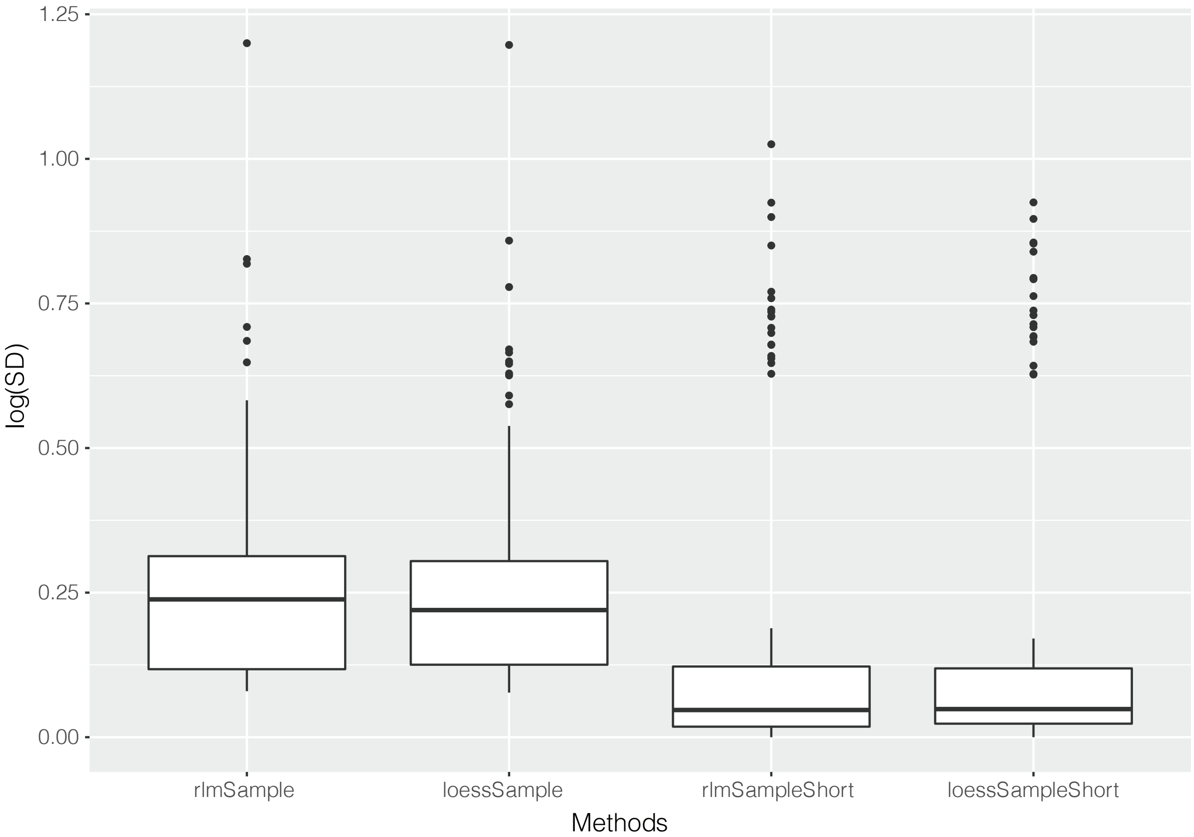

Supplementary Figure 4. A comparison of intra-batch normalisation methods with and without RUV using short replicates (*n*=115 sample replicates for each method). The y-axis is a natural log transformed SD. The box indicates quartiles and the whiskers indicate the rest of the distribution, with outliers shown as dots. Source data are provided as a Source data file.

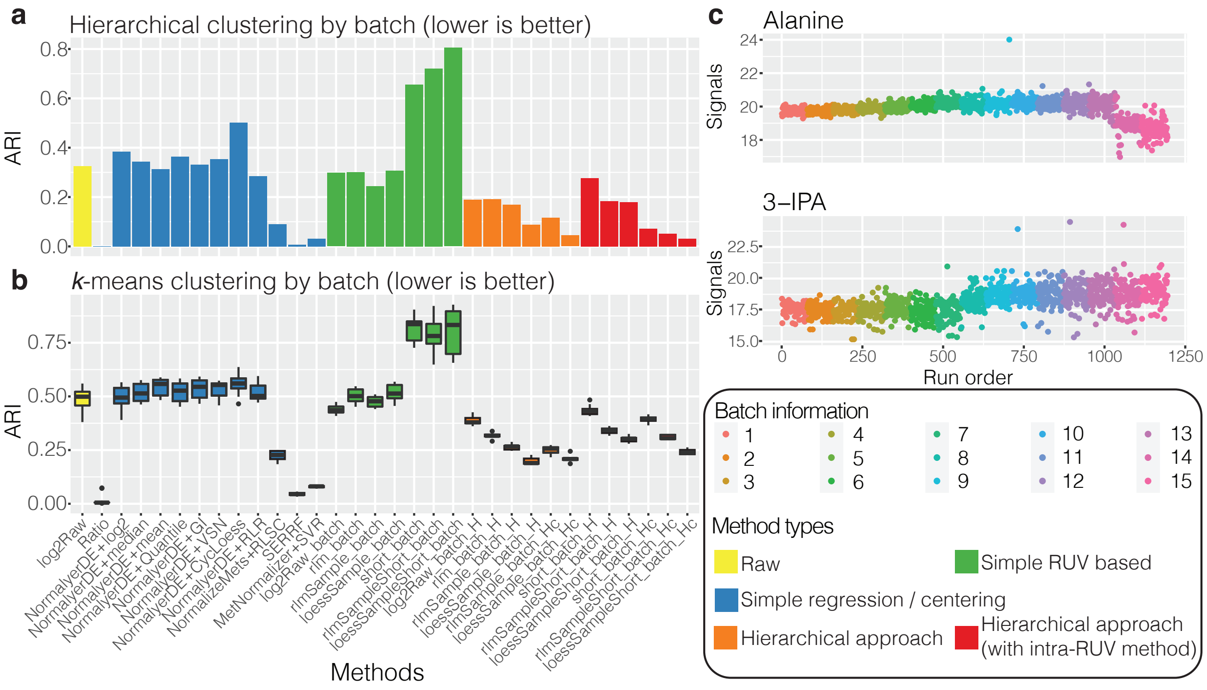

Supplementary Figure 5. A comparison of different normalization approaches in removal of batch effect. a A bar plot of Adjusted Rand Indices (ARI) which compares the concordance of hierarchical clustering to the known batch information. Higher ARIs indicate clusters driven by batch effects; thus lower ARIs indicates better normalisation. **b** Similar to **a**, a boxplot of ARI concordance of 10 permutations of *k*-means clustering to the known batch information. **c** A sample run plot for the metabolites alanine and 3-indolepropionic acid with loessSampleAllShort_batch_Hc normalised data. The samples are coloured by batch number. In **b**, the box indicates quartiles and the whiskers indicate the rest of the distribution, with outliers shown as dots. Source data are provided as a Source data file.

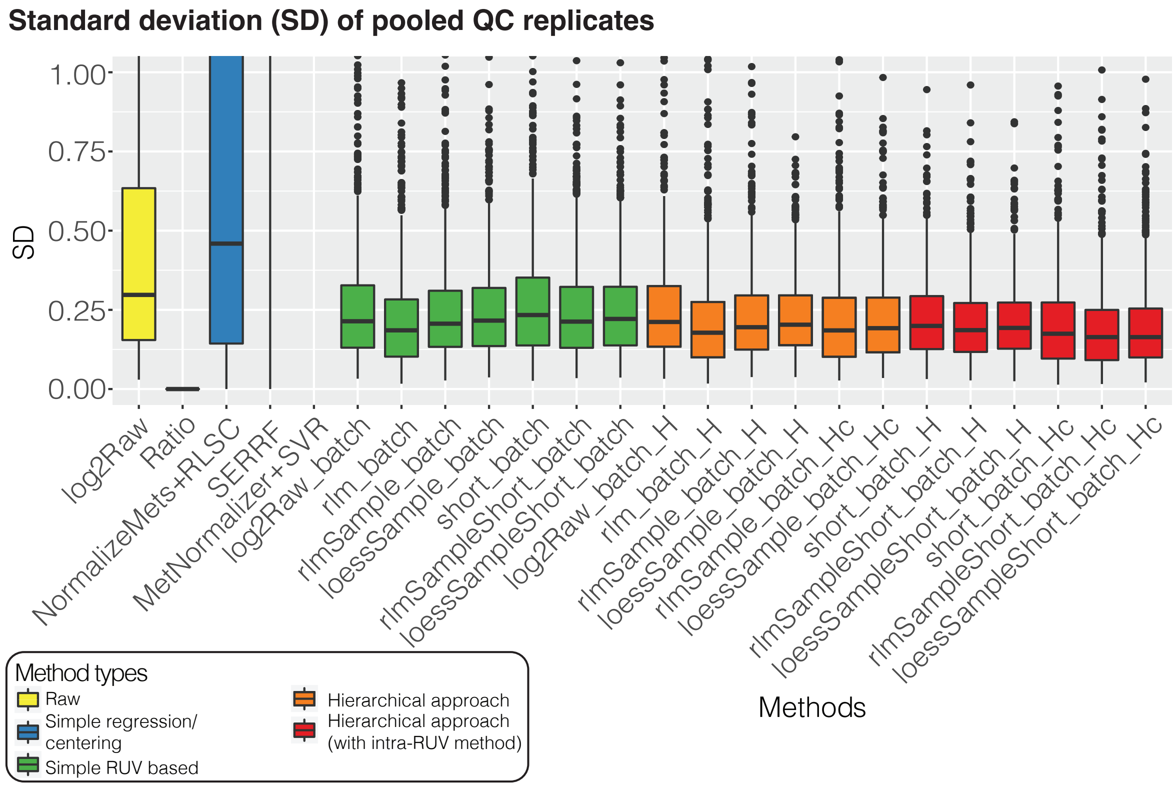

Supplementary Figure 6. **Boxplots of Pooled QC sample replicates standard deviations**. The boxes are coloured by the approach taken to normalize the data. The y-axis of the plot is restricted to a range between 0 to 1 to highlight the differences between the majority of the methods. The lower SD (y-axis) indicates better performance. SERRF (*n*=1185) and MetNormalizer+SVR's (*n*=1185) median sample replicate SD was greater than 1 and thus is not shown. The distribution of NMets_RLSC is plotted with *n*=1155 and other methods including raw (yellow), ratio, simple RUV based (green) and hierarchical approaches (orange and red) are plotted with *n*=795. The box indicates quartiles and the whiskers indicate the rest of the distribution, with outliers shown as dots. Source data are provided as a Source data file.

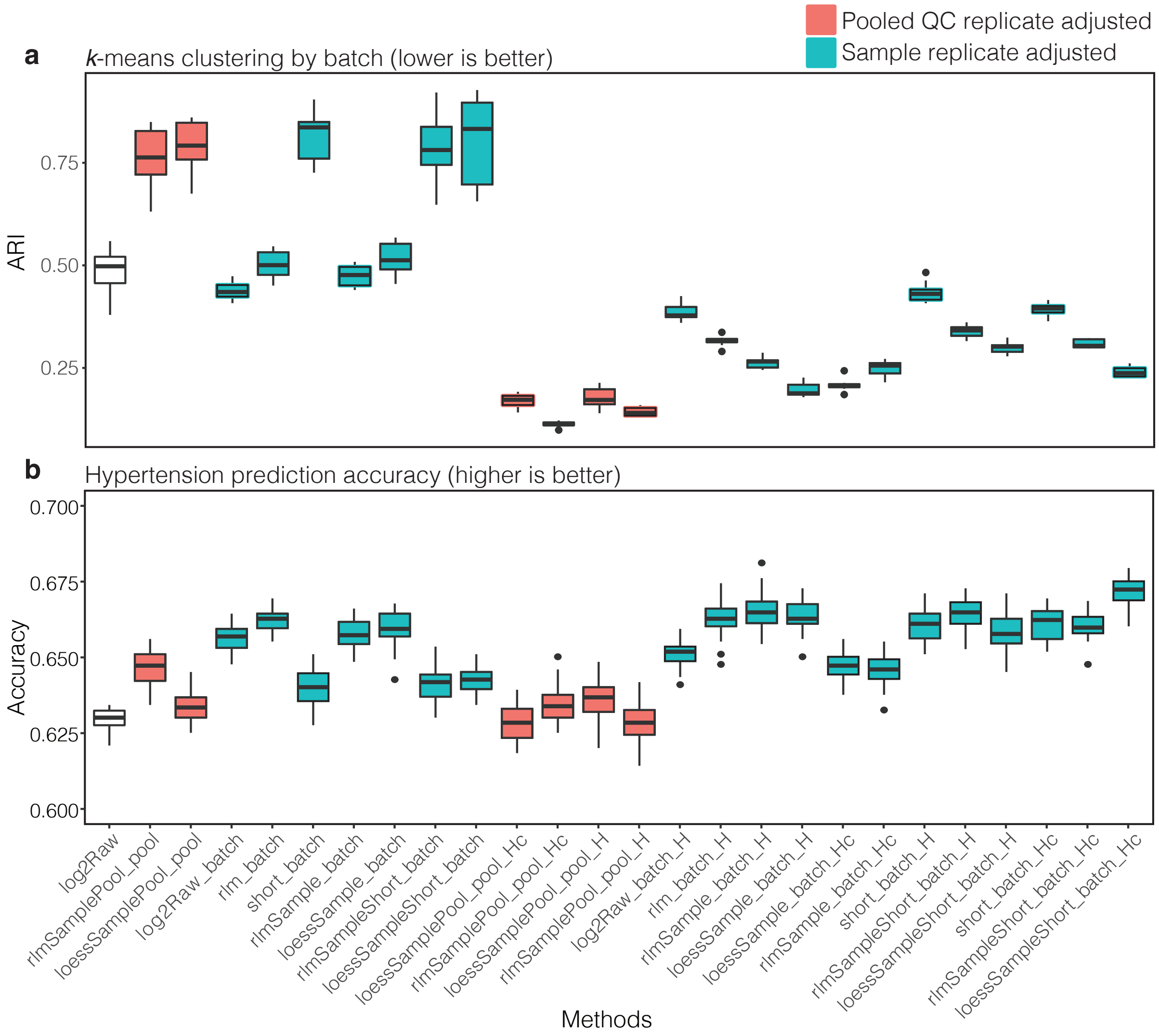

Supplementary Figure 7. A comparison of RUV based normalization approaches using sample replicates versus using pooled QC replicates. a Boxplots of clustering ARIs (*n*=10 for all methods) compared to batch with pooled QC replicate adjusted RUV and sample replicate RUV adjusted data. **b** Boxplots of hypertension prediction accuracies (*n*=30 for all methods) for the same methods as in **a**. In **a-b**, the box indicates quartiles and the whiskers indicate the rest of the distribution, with outliers shown as dots. Source data are provided as a Source data file.

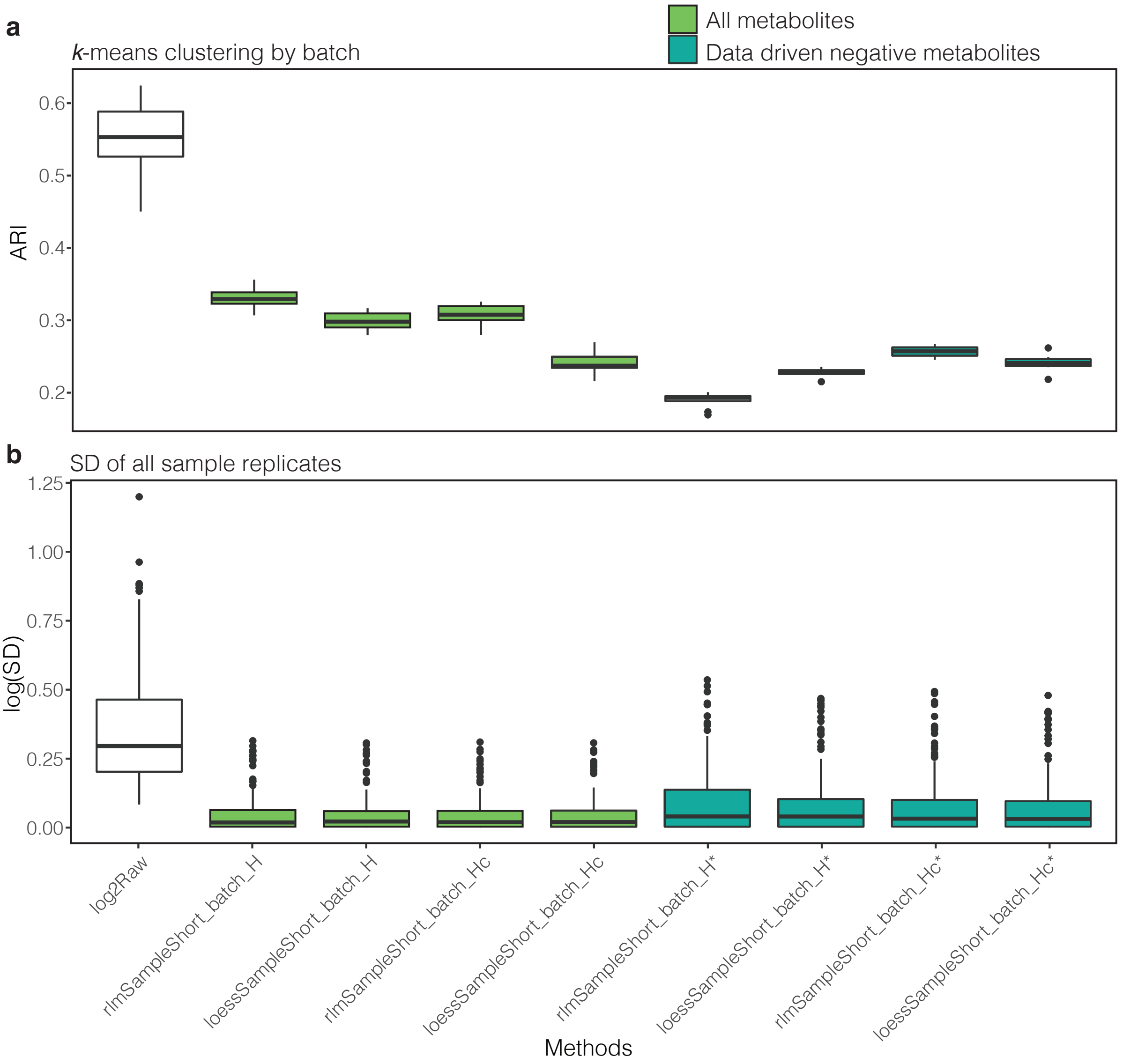

Supplementary Figure 8. **Comparison of results using different sets of negative controls in RUV. a** Boxplot comparison of ARIs (n=10 repeats for all methods) from *k*-means batch clustering. **b** Boxplots of standard deviations from all sample replicates (n=175 sample replicates for all methods) for the normalisation methods with all metabolites and data driven negative metabolites as negative controls in RUV. The y-axis is a natural log transformed SD. In **a-b**, the box indicates quartiles and the whiskers indicate the rest of the distribution, with outliers shown as dots. Source data are provided as a Source data file.

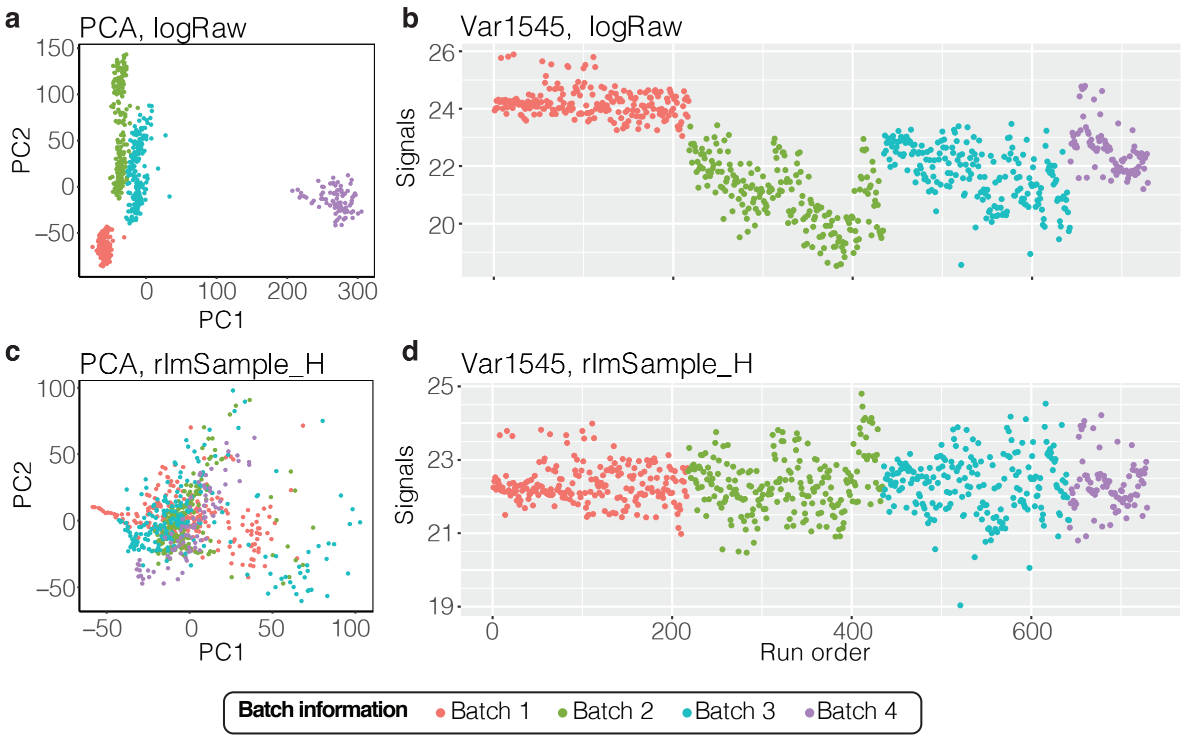

Supplementary Figure 9. **Comparison of unnormalized and hRUV normalized untargeted LC/MS metabolomics dataset.** This figure illustrates the capability of hRUV where we applied hRUV normalization to an untargeted LC/MS metabolomics from Deng et al[1]. The data contains 729 samples with 6402 features from four batches with no sample replicates. **a** The PCA plot shows separation of the data by batch information in the raw data. **b** The run plot of raw data demonstrates different types of signal drifts and a strong batch effect in a feature Var1545. **c** The PCA plot of hRUV normalized (rlmSample_H) data, here we do not observe separation by batch. **d** The run plot of hRUV normalized (rlmSample_H) data demonstrates removal of signal drifts and batch effect. Source data are provided as a Source Data file.

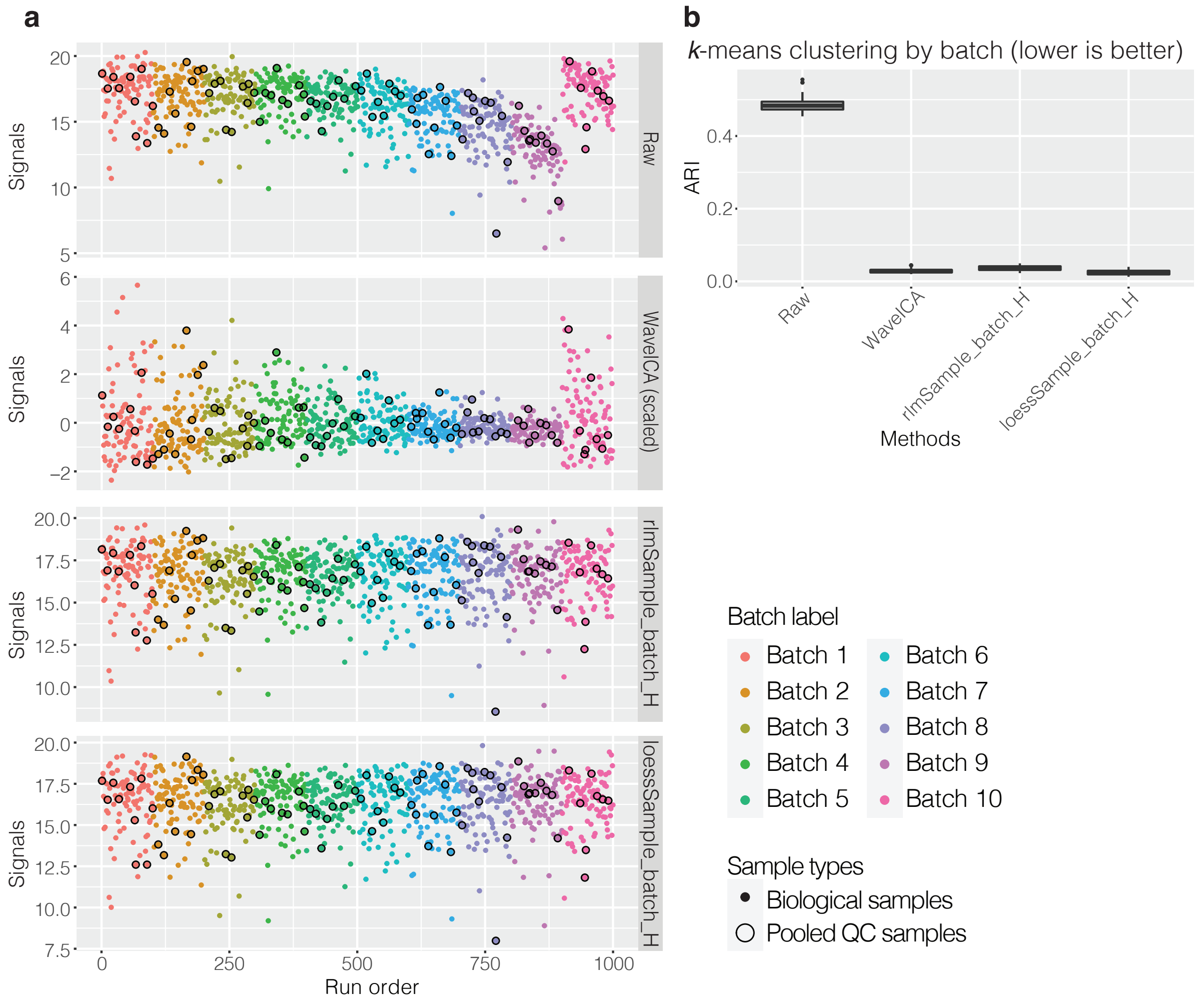

Supplementary Figure 10. Comparison of hRUV on simulated untargeted metabolomics data. This large-scale simulated untargeted metabolomics data consists of 1000 samples including 82 QC samples with 5000 features separated into 10 batches. Each batch has total of 100 samples including 8 pooled QC samples, 5 batch replicate samples and 8 pairs of short replicates. The data is generated using an R package `mzrtsim`[2] with our additional modification to simulate sample replicates. We have set the 99% of the features to have varying degree of batch effect defined as random, monotonic and block noise, and 3% of features to be condition specific features. **a** A comparison of one of the features run plot in a simulated untargeted metabolomics data. Each marker indicates samples colored by the batch information. The signals of a raw data (first row) have a clear sign of batch effect and a signal drift. The WaveICA[1] (second row) is a batch correction method specifically for an untargeted data, but it is not able to remove all unwanted variation. The variability of signal changes across the runs and a spike in variability for batch 10. hRUV applied methods (third and fourth row) does not show a sign of batch effect in the run plot. **b** A boxplot comparison of ARIs from *k*-means batch clustering (*n*=30 repeats for all methods). The boxplot demonstrates the removal of batch effect as the ARI of hRUV methods and WaveICA methods are close to zero. In **b**, the box indicates quartiles and the whiskers indicate the rest of the distribution, with outliers shown as dots. Source data are provided as a Source data file.

Supplementary Tables

HPLC gradient parameters

Time (min)	Flow Rate (μl/min)	Mobile Phase A(%)	Mobile Phase B(%)
0	250	5	95
0.5	250	5	95
6	250	60	40
9	250	60	40
10	250	5	95
11	400	5	95
23.5	400	5	95
24.5	250	5	95
25	250	5	95

Supplementary Table 1. A HPLC gradient parameters for mobile phase A and B.

Supplementary Note 1:

A typical workflow consists of three components and please see https://sydneybiox.github.io/hRUV/ for more details.

Component A: Preprocessing
The preprocessing steps involved before applying hRUV includes standard filtering, imputation of the data and to get an intersecting set of metabolites across all batches. A $\log_2$ transformed raw signal matrix as a `SummarizedExperiment` object is expected in our hRUV package to perform in a single line of command in `clean` function. The function will remove metabolites with high proportion of missing values and perform *k*-nearest neighbour imputation. A set of non-intersecting metabolites across all batches are then removed.

```
> dat_list = hRUV::clean(dat_list, threshold = 0.5, method =
"intersect", assay = "logRaw", newAssay, "rawImpute")
```

Example code above will remove metabolites with over 50% missing value across a batch and perform imputation on a "logRaw" assay, where `dat_list`, is a list of `SummarizedExperiment` object where each element in the list indicates a batch.

Component B: Intra-batch normalization
In intra-batch normalization, a smoothing function is fitted to correct for signal drift and an additional RUV step can be applied if necessary to further correct for unwanted intra-batch variations. List of parameter options for intra-batch normalization in hruv include:

- "loess" - Signal drift correction by fitting a loess curve.
- "rlm" - Signal drift correction by fitting a robust linear model.
- "loessShort" - Signal drift correction by fitting a loess curve. Then perform RUV-III within each batch.
- "rlmShort" - Signal drift correction by fitting a robust linear model. Then perform RUV-III within each batch.

Component C: Inter-batch normalization
Inter-batch normalization involves progression RUV normalization in a tree-structure using batch replicate samples. List of options for inter-batch normalization

- "concatenate" - Perform hierarchical RUV-III in a concatenate tree structure.
- "balanced" - Perform hierarchical RUV-III in a balanced tree structure.

Note. Component B and C of hRUV can be performed with a single line of command in R with `hruv` function.

```
> dat = hruv(
      dat_list = dat_list,
      assay = "rawImpute",
```

```
    intra = "loessShort",
    inter = "concatenate",
    intra_k = 5, inter_k = 5,
    pCtlName = "biological_sample",
    negCtl = NULL,
    intra_rep = "short_replicate",
    inter_rep = "batch_replicate"
)
```

In the example code above, given that `dat_list` is a list of `SummarizedExperiment` objects from Component A, the function will perform intra-batch normalization (Component B) by fitting loess curve to "biological_sample" and perform RUV-III within batch using "short_replicate" samples. The inter-batch normalization (Component C) is then performed in a "concatenate" tree structure with "batch_replicate" samples. Further details and examples of the functions are available in the hRUV package documentations.

Supplementary References

1. Deng, K. *et al.* WaveICA: A novel algorithm to remove batch effects for large-scale untargeted metabolomics data based on wavelet analysis. *Anal. Chim. Acta* **1061**, 60–69 (2019).
2. Yu, M., Roszkowska, A. & Pawliszyn, J. Simulation-based comprehensive study of batch effects in metabolomics studies. *bioRxiv* 2019.12.16.878637 (2019) doi:10.1101/2019.12.16.878637.
