# Supplementary material for: A hierarchical approach to removal of unwanted variation for large-scale metabolomics data
Source: Nat Commun. 2021 Aug 17;12:4992. doi: 10.1038/s41467-021-25210-5 (PMC8371158; doi:10.1038/s41467-021-25210-5)
Supplement: Supplementary file 2 — Description of Additional Supplementary Files [file 41467_2021_25210_MOESM2_ESM.pdf]

Description of Additional Supplementary Files

Supplementary Data 1. A list of all the metabolites measured and their technical details. The summary statistics are measured from log2 transformed raw data.

Supplementary Data 2. The batch sample arrangement design represented as a plate format used in the study to embed intra-batch and inter-batch sample replicates. There are 8 rows and 11 columns per batch, samples marked with "*" indicates intra-batch sample replicates and "***" indicates inter-batch sample replicates.
