# Supplementary material for: A hierarchical approach to removal of unwanted variation for large-scale metabolomics data
Source: Nat Commun. 2021 Aug 17;12:4992. doi: 10.1038/s41467-021-25210-5 (PMC8371158; doi:10.1038/s41467-021-25210-5)
Supplement: Supplementary file 5 — Reporting summary [file 41467_2021_25210_MOESM5_ESM.pdf]

nature research

Corresponding author(s): Jean YH Yang

Last updated by author(s): Jul 7, 2021

Reporting Summary

Nature Research wishes to improve the reproducibility of the work that we publish. This form provides structure for consistency and transparency in reporting. For further information on Nature Research policies, see our Editorial Policies and the Editorial Policy Checklist.

Statistics

For all statistical analyses, confirm that the following items are present in the figure legend, table legend, main text, or Methods section.

n/a	Confirmed	
☐	☒	The exact sample size (n) for each experimental group/condition, given as a discrete number and unit of measurement
☐	☒	A statement on whether measurements were taken from distinct samples or whether the same sample was measured repeatedly
☐	☒	The statistical test(s) used AND whether they are one- or two-sided<br>*Only common tests should be described solely by name; describe more complex techniques in the Methods section.*
☒	☐	A description of all covariates tested
☒	☐	A description of any assumptions or corrections, such as tests of normality and adjustment for multiple comparisons
☐	☒	A full description of the statistical parameters including central tendency (e.g. means) or other basic estimates (e.g. regression coefficient) AND variation (e.g. standard deviation) or associated estimates of uncertainty (e.g. confidence intervals)
☐	☒	For null hypothesis testing, the test statistic (e.g. F, t, r) with confidence intervals, effect sizes, degrees of freedom and P value noted<br>*Give P values as exact values whenever suitable.*
☒	☐	For Bayesian analysis, information on the choice of priors and Markov chain Monte Carlo settings
☐	☒	For hierarchical and complex designs, identification of the appropriate level for tests and full reporting of outcomes
☐	☒	Estimates of effect sizes (e.g. Cohen's d, Pearson's r), indicating how they were calculated

Our web collection on statistics for biologists contains articles on many of the points above.

Software and code

Policy information about availability of computer code

Data collection

Data was acquired using Analyst 1.6.2 Build 8489
Data was integrated using MultiQuant 3.0.3

Data analysis

The development of the R package and data analysis was performed with R version 4.0.3 (https://www.R-project.org/). The package developed are available at https://github.com/SydneyBioX/hRUV.
In addition to the base R package, the list of R packages used include:
- ruv [version 0.9.7.1]
- MetNormalizer [version 1.3.02]
- NormalyzerDE [version 1.7.0]
- NormalizeMets [version 0.24]
- limma [version 3.46]
- ClassifyR [version]
- DMwR2 [version 0.0.2]
- SummarizedExperiment [version 1.20.0]
- MASS [version 7.3-53.1]
- e1071 [version 1.7-4]
- ggplot2 [version 3.3.3]
A shiny app [https://slfan.shinyapps.io/ShinySERRF/] was also used for a benchmark.

For manuscripts utilizing custom algorithms or software that are central to the research but not yet described in published literature, software must be made available to editors and reviewers. We strongly encourage code deposition in a community repository (e.g. GitHub). See the Nature Research guidelines for submitting code & software for further information.

Data

Policy information about availability of data

All manuscripts must include a data availability statement. This statement should provide the following information, where applicable:

- Accession codes, unique identifiers, or web links for publicly available datasets
- A list of figures that have associated raw data
- A description of any restrictions on data availability

The raw metabolomics data generated in this study have been deposited in the MetaboLights under accession code MTBLS2483 [https://www.ebi.ac.uk/metabolights/index]. The processed metabolomics data are available at GitHub repository [https://github.com/SydneyBioX/BioHEART_metabolomics]. The sample order design data generated in this study are provided in the Supplementary Data 2. The figure data generated in this study are provided in the Source Data file.

Field-specific reporting

Please select the one below that is the best fit for your research. If you are not sure, read the appropriate sections before making your selection.

☒ Life sciences ☐ Behavioural & social sciences ☐ Ecological, evolutionary & environmental sciences

For a reference copy of the document with all sections, see nature.com/documents/nr-reporting-summary-flat.pdf

Life sciences study design

All studies must disclose on these points even when the disclosure is negative.

Sample size	One aspect of our study (the discovery cohort from BioHEART-CT study) was performed with 1002 unique samples. Our manuscript is methodological development paper and the sample size calculation is not relevant in our study design.
Data exclusions	Data were not excluded from analysis
Replication	Study replication is not relevant to this study. The proposed method was tested on independent dataset.
Randomization	Experiment sample replicate selection was randomly generated using software R.
Blinding	Blinding was not relevant to this study, which was designed to develop and evaluate normalisation method for large-scale metabolomics data. Such methods cannot be developed while blind to the results.

Reporting for specific materials, systems and methods

We require information from authors about some types of materials, experimental systems and methods used in many studies. Here, indicate whether each material, system or method listed is relevant to your study. If you are not sure if a list item applies to your research, read the appropriate section before selecting a response.

Materials & experimental systems

n/a	Involved in the study	
☒	☐	Antibodies
☒	☐	Eukaryotic cell lines
☒	☐	Palaeontology and archaeology
☒	☐	Animals and other organisms
☐	☒	Human research participants
☒	☐	Clinical data
☒	☐	Dual use research of concern

Methods

n/a	Involved in the study	
☒	☐	ChIP-seq
☒	☐	Flow cytometry
☒	☐	MRI-based neuroimaging

Human research participants

Policy information about studies involving human research participants

Population characteristics	The samples used were from the discovery cohort of the BioHEART-CT, a multicentre, longitudinal, prospective cohort study of patients undergoing a Computed tomography coronary angiography (CTCA) for investigation of suspected Cardiovascular disease. The BioHEART-CT discovery cohort utilised for this analysis included the first 1002 patients recruited to the BioHEART-CT study who had technically adequate CTCAs, sufficient stored blood samples for all planned biomarker discovery platforms, and who did not have a cardiac stent in situ or a prior history of coronary artery bypass surgery.<br><br>The BioHEART-CT discovery cohort of 1002 patients have a median age of 62 with 447 female patients and an average BMI (kg/m2) of 26.9. In addition, 87% of the cohort have been diagnosed with diabetes mellitus, 38.9% of the cohort with hypertension, 59.9% of the cohort with hypercholesterolaemia and 20.7% of the cohort with a significant smoking history. The population cohort are from various heritage backgrounds, 87% European, 6.2% Asian, 2.1% Middle Eastern, 1.7% Indian, 0.8% African, 0.5% Polynesian, 0.4% Hispanic, 0.4% Indigenous Australian and 0.9% missing.
Recruitment	Patients undergoing clinically indicated CTCA for suspected coronary artery disease were recruited from multiple sites in Sydney, Australia. The inclusion criteria for BioHEART-CT study are: patients aged 18 or older who had been referred for investigation of suspected CAD by CTCA, and who were willing and able to provide informed consent. Patients who were highly dependent on medical care who were unable to provide informed consent, as well as patients who were unwilling or unable to participate in ongoing follow-up were excluded.<br><br>Potential self-selection bias may have an impact in future analysis (after normalization) but it is not relevant for this paper. The focus of the study is to remove unwanted variation (bias) that comes from metabolomics profiling of these samples.
Ethics oversight	The data reported in this study is from the discovery cohort and has been approved by the study investigators. The study protocol and design has been approved by the Northern Sydney Local Health District Human Research Ethics Committee (HREC/17/HAWKE/343) and all participants provided informed, written consent. The current analysis is inline with this original approval and consent. BioHEART is a registered Australian and New Zealand Clinical Trial (ACTRN12618001322224).

Note that full information on the approval of the study protocol must also be provided in the manuscript.
